# Predictors of cognitive functioning trajectories among older Americans: A new investigation covering 20 years of age- and non-age-related cognitive change

**DOI:** 10.1371/journal.pone.0281139

**Published:** 2023-02-08

**Authors:** Hui Zheng, Kathleen Cagney, Yoonyoung Choi

**Affiliations:** 1 Department of Sociology, Institute for Population Research, The Ohio State University, Columbus, Ohio, United States of America; 2 Department of Sociology, Institute for Social Research, University of Michigan, Ann Arbor, Michigan, United States of America; IUPUI: Indiana University Purdue University at Indianapolis, UNITED STATES

## Abstract

Despite the extensive study of predictors of cognitive decline in older age, a key uncertainty is how much these predictors explain both the intercept and age- and non-age-related change in cognitive functioning (CF). We examined the contribution of a broad range of life course determinants to CF trajectories. Data came from 7,068 participants in the 1996–2016 Health and Retirement Study. CF was measured as a summary score on a 27-point cognitive battery of items. We estimated multilevel growth curve models to examine the CF trajectories in individuals ages 54–85. We found that the variation in CF level at age 54 was three times as much as the variation in age slope. All the observed individual predictors explained 38% of the variation in CF at age 54. Personal education was the most important predictor (25%), followed by race, household wealth and income, parental education, occupation, and depression. The contributions of activity limitations, chronic diseases, health behaviors (obesity, smoking, vigorous activity), childhood conditions (childhood health, nutrition, financial situation), gender, marital status, and religion were rather small (<5%). Even though the age slope varied with many adulthood factors, they only explained 5.6% of the between-person variation in age slope. Moreover, age explained 23% of within-person variation in CF from age 54 to 85. The rest non-age-related within-person variation could not be explained by the observed time-varying factors. These findings suggest that future research is urgently needed to discover the main determinants of the slope of cognitive decline to slow down the progression of cognitive impairment and dementia.

## Introduction

Dementia, described in the early 1980s as “The Silent Epidemic” [1], is expected to become a major disease in most countries around the world as the aging population continues to grow. The number of individuals with dementia is estimated to increase to 131.5 million by 2050 [2]. With the aging U.S. population, the number of Americans living with Alzheimer’s is projected to nearly triple from 5.8 million to 13.8 million between now and 2050 [3]. This prospect will likely create a substantial burden for Medicare, the healthcare system, and consequently the economy. Dementia, however, only accounts for 41% of cognitive decline among the elderly [4]. Of the 41%, 30–34% are caused by Alzheimer’s disease, 1–3% are caused by cerebrovascular disease, and 4–8% are caused by Lewy body disease [4]. The remaining 59% of cognitive decline is not explained by these neuropathologic indices [4]. Therefore, this study focuses on overall cognitive function and cognitive decline like some prior studies [5], which has a broad implication on population brain health because cognitive decline is pervasive in older adults, even among those without dementia.

Low cognitive functioning (CF) and cognitive decline are associated with an increased risk of poor quality of life, morbidity, and mortality [6, 7]. It is critical to discover why some individuals’ cognitive abilities are better than others, and why their cognitive declines are slower. Solving this problem has important ramifications for policymakers and medical interventions to target the causes that are most influential on cognitive trajectories. The notion of the level and trajectory of cognitive functioning is also consistent with social scientists’ core interests in how inequality unfolds over the life course. “Cumulative advantage/disadvantage” theory posits that disparities in health between more and less advantaged groups increase over the life course due to the cumulative health benefits of advantaged resources [8–10]. According to this theory, we would expect individuals with more advantaged early-life and adulthood characteristics experience both better cognitive functioning and a slower decline in cognition in later life than those in more adverse situations.

While life course determinants of CF trajectories have been extensively studied and many risk and protective factors have been proposed, a key uncertainty continues to be the relative contribution of these factors to the variation in both the level and slope (or trajectory) of decline in CF [5, 7]. The factors proposed by social and behavioral scientists and epidemiological researchers span genes, early life nutrition, diseases, family background, adulthood socioeconomic status, psychosocial factors, physical and bio-behavioral factors, health, and lifestyle [11–14]. However, findings from these studies are sometimes inconsistent or even contradictory regarding the effects of these contributors to either the level or the slope of CF, meaning that these topics remain the subject of debate [5, 7].

Studies generally conclude that factors such as smoking status [15, 16], depressive disorders [17–19], diabetes mellitus [20], metabolic syndrome [21], and the APOE e4 genotype [22, 23] are associated with more cognitive decline, while better physical health [24], physical activity [25, 26], Mediterranean diet [27], and cognitive training [28] are protective factors.

Research on the relationship between sociodemographic factors, such as sex, socioeconomic status, education, marriage, and cognitive decline, however, has yielded mixed results [5]. For example, some studies report a steeper decline in CF among men than women [29], while other studies do not find gender differences [30]. Childhood and adulthood socioeconomic conditions contribute to the initial level of CF [31–38] but do not affect age-related cognitive decline [13, 39–44], which is inconsistent with what the “cumulative advantage/disadvantage” theory would predict.

These mixed findings often come from different datasets, research designs, time periods, and countries [7]. They may also stem from the fact that each individual study only assessed a limited number of predictors without accounting for intercorrelations with unobserved confounders, with a few notable exceptions [5, 13], which, however, have either a narrow-age cohort or short follow-up. Moreover, most prior studies have primarily focused on *between-person* differences in the level and age slope of CF. The age slope of CF refers to the trajectory of CF over ages. Prior studies have focused on how the age slope may vary across individuals by their sociodemographic, behavioral, or other factors, which may cause a faster or slower decline in CF over ages. Few of them have examined *within-person* non-age-related cognitive variability and what factors may shape this within-person heterogeneity. Non-age-related cognitive variability implies the uncertainty in cognitive change within an individual’s life course that cannot be explained by the aging process. It reflects the substantial heterogeneity in the pattern of cognitive trajectory in the population that is beyond the normally studied between-person variation in the age trajectory of CF. Therefore, it is important to consider within-person non-age-related cognitive variability in addition to between-person variation in the level and age slope of CF in order to obtain a complete picture of CF trajectories in the population.

In this study, we used large nationally representative longitudinal data with a long follow-up period and a broad range of factors to determine essential contributors to CF trajectories (level, age- and non-age-related slope). Different from prior studies that have focused on a small number of predictors and the causal pathway among these predictors, we took a step back and investigated a wide range of factors and their relative contributions to the CF trajectories. These analyses can avoid mixed findings due to different datasets, account for the intercorrelations among the predictors, and effectively estimate both the statistical significance and the substantive contribution of these predictors. More specifically, we used the U.S. Health and Retirement Study (HRS) with 20 years of follow-up to model the pattern and heterogeneity of CF trajectories and estimated a wide range of potential predictors, that included childhood conditions (childhood health, nutrition, financial situation, and parental education), adult socioeconomic status (education, occupation, household wealth, and household income), psychosocial factors (current marital status, marital history, children, religion, and depression), physical status (activity limitations, and chronic diseases), and bio-behavioral factors (body mass index (BMI), smoking status, and vigorous activity).

## Materials and methods

### Data and participants

We used data from the initial HRS cohort (born 1931–1941) from the years 1996 to 2016. The HRS is a multi-cohort study conducted by the Institute for Social Research at the University of Michigan and primarily sponsored by the National Institute on Aging. It is a nationally representative, biennial, longitudinal survey of persons ages 51 or older since 1992. Refreshment cohorts of ages 51–56 enter the survey every six years. The first two waves were not used because they employed different cognition measurements. The refreshment cohorts were not used because they were relatively young and had limited observations of CF with which to model the entire trajectory above age 50. We focused on the initial cohort who were 54–65 years old (mean age 59, SD = 3.2) in 1996 and aged to 74–85 (mean age 79, SD = 3.1) in 2016, which enabled us to model the CF trajectory from age 54 to age 85. We made use of imputed cognitive measures provided by HRS [45]. Raw data were not used because they missed a large number of respondents with dementia. Our original sample included 9,568 individuals ages 54 or older and 74,087 observations from 1996 to 2016. Dropping missing data on cognitive functioning (interviews completed by proxy respondents were excluded because they did not take a cognitive test) left 9,256 individuals with 68,898 observations. Dropping individuals with missing data on the explanatory variables, we reached a final sample size of 7,068 individuals with 54,400 observations. Supporting Information 1 in S1 File shows the flowchart of the study sample. The sample included does not display a significant difference in the observed characteristics from those excluded. All relevant data are publicly available from the OSF repository (https://osf.io/em7kj/).

### Measures

Table 1 shows the detailed descriptive statistics of the sample based on all the observations (54,400 observations) from 1996 to 2016. CF was measured as a summary score on a 27-point cognitive battery of items: immediate and delayed word recall scores (0–20 points), a serial sevens subtraction test score (0–5 points), and backward counting from 20 (0–2 points). The summary cognition score ranged from 0 to 27. A greater number of points reflected better CF. Q-Q plots found this variable had a normal distribution with thin tails. In some analyses, we used the Langa-Weir specification to operationalize three cognition statuses: a total CF score of 0–6 points is labeled as “demented,” 7–11 as “cognitively impaired but not demented (CIND),” and 12–27 as “normal” [46]. This dementia classification has been verified by a clinically evaluated neuropsychological examination on a subsample of the HRS called the Aging Demographics and Memory Study [46].

**Table 1:** Descriptive statistics of sample (54,400 observations).

**Variable**	**Description**	**n**	**Mean (SD) or Prop.**
Cognition score	Summary scores of immediate and delayed word recall scores (0–20 points), a serial sevens subtraction test score (0–5 points), and backwards counting from 20 (0–2 points). The summary cognition score ranges from 0 to 27.	54,400	15.67 (4.37)
Age	Age at interview	54,400	67.98 (6.80)
Male	Sex	54,400	44.51%
Race/Ethnicity	Race / ethnicity: with Hispanic origin	54,400	
Non-Hispanic White			75.51%
Non-Hispanic Black			14.53%
Hispanic			8.03%
Other			1.93%
**Childhood Conditions**			
Childhood health	Self-rated health while the respondent was growing up, before age 16.	54,400	
Poor			1.42%
Fair			4.89%
Above average			17.40%
Very good			25.03%
Excellent			51.26%
Height in inches	First reported adult height, as a proxy for childhood nutrition	54,400	66.87 (3.94)
Childhood finance	Self-rated family financial situation while the respondents were growing up, before age 16	54,400	
Pretty well off			5.63%
About average			63.88%
Poor			30.49%
Parental education	Mother’s or father’s number of years of education, whichever are larger	54,400	10.23 (3.63)
**Variable**	**Description**	**n**	**Mean (SD) or Prop.**
**Adult Socioeconomic Status**			
Education	Years of education	54,400	12.63 (3.00)
College degree	Whether the respondent has a college degree (1 = yes)	54,400	21.99%
Occupation	Occupation for job with longest reported tenure	54,400	
White collar			36.47%
Blue collar			63.53%
Household income	Total household income for the last calendar year. It is the sum of respondent and spouse earnings, pensions and annuities, SSI and Social Security Disability, Social Security retirement, unemployment and workers compensation, other government transfers, household capital income and other income. It is adjusted for household size by dividing by the square root of the number of people in the household.	54,400	
Below 25%			20.35%
25–75%			55.11%
Above 75%			24.54%
Household wealth	Total of all assets, including residence, net value of real estate, vehicles, businesses, individual retirement accounts, Keogh accounts, stocks, mutual funds, investment trusts, checking, savings, money market accounts, certificates of deposit, government savings bonds, treasury bills, bonds, and bond funds, and all other savings, less sum of all mortgages, other home loans, and other debt. It is adjusted for household size by dividing by the square root of the number of people in the household.	54,400	
Below 25%			19.04%
25–75%			51.41%
Above 75%			29.56%
**Variable**	**Description**	**n**	**Mean (SD) or Prop.**
**Adult Psychosocial Factors**			
Current marital status	Current marital status: with partnership	54,400	
Married			64.63%
Partnered			2.65%
Separated or divorced			12.59%
Widowed			16.79%
Never married			3.33%
Number of marriages	Marital history: number of marriages	54,400	
0			3.36%
1			67.21%
2			22.33%
3			5.32%
4+			1.78%
Number of children	Number of living children	54,400	3.38 (2.09)
Religion	Religious preference	54,400	
Protestant			66.43%
Catholic			26.99%
Jewish			1.67%
None			4.28%
Other			0.63%
CESD	Summary score of eight measures: felt depressed, everything was an effort, sleep was restless, was happy, felt lonely, sad, could not get going, and enjoyed life. Each measure is a yes/no indicator of the respondent’s feelings much of the time over the week prior to the interview. CESD score ranges from 0 to 8.	54,400	1.29 (1.84)
**Variable**	**Description**	**n**	**Mean (SD) or Prop.**
**Adult Bio-behaviors and Diseases**			
Body Mass Index (BMI)		54,400	
Underweight	BMI<18.5		1.22%
Normal weight	18.5< = BMI<25		29.17%
Overweight	25< = BMI<30		39.99%
Obese I	30< = BMI<35		20.28%
Obese II/III	BMI> = 35		9.34%
Ever smoker	Has the respondent ever smoked cigarettes?	54,400	60.60%
Current smoker	Does the respondent smoke cigarettes now?	54,400	13.74%
Vigorous Activity	Vigorous physical activity or sports at least once per week.	54,400	40.25%
Number of chronic diseases	Summary score of ever having the following chronic diseases: psychiatric problem, stroke, heart disease, high blood pressure, diabetes, lung disease, cancer, and arthritis.	54,400	1.99 (1.42)
High blood pressure	Ever had high blood pressure		55.21%
Diabetes	Ever had diabetes		19.58%
Lung disease	Ever had lung disease		9.26%
Heart disease	Ever had heart problems		23.04%
Stroke	Ever had stroke		6.70%
Psychiatric problem	Ever had psychiatric problems		11.40%
Cancer	Ever had cancer		14.36%
Arthritis	Ever had arthritis		58.97%
ADL	Activities of daily living that the subject cannot do or has difficulty doing: bathing, eating, dressing, walking across a room, and getting in or out of bed	54,400	0.23 (0.72)
IADL	Instrumental activities of daily living that the subject cannot do or has difficulty doing: using a telephone, taking medication, handling money, shopping, preparing meals	54,400	0.16 (0.58)

HRS data have very rich information on childhood conditions, and adulthood socioeconomic, psychosocial, physical, and bio-behavioral factors, which enabled us to conduct a comprehensive examination of the relative contribution of life course factors to the intercept and slope of CF. Childhood conditions included childhood health, nutrition, financial situation, and parental education. Childhood health was the respondent’s self-rated health while he or she was growing up, which was reported on a 5-point scale: 1 (poor), 2 (fair), 3 (above average), 4 (very good), or 5 (excellent). We used adult height as a marker of a child’s early environment. The association between height at age 3 and height in adulthood is strong, so adult height provides a good marker of one’s early life nutrition and health environment [47]. Respondents reported their heights in Waves 1 and 2. Respondents who were not surveyed in Waves 1 and 2 reported their heights when they were first interviewed. Even though height might shrink in older ages, the respondents were still relatively young when they were first interviewed, and self-reports of height may be less likely to reflect age-related shrinkage than measured height [47]. Childhood financial situation was assessed on a 3-point scale: 1 (poor), 2 (about average), or 3 (pretty well off). Parental education was the mother’s or father’s number of years of education, whichever is greater.

Adult socioeconomic conditions included education, occupation, household wealth, and household income. Adulthood education was measured as either years of education or the highest degree attained. Occupation was the job with the longest reported tenure, which was further categorized as white-collar or blue-collar. Household wealth was the sum of both financial and nonfinancial assets (e.g., homes, vehicles, stocks) minus the sum of all debts (e.g., mortgages). Household income was the sum of respondent and spouse earnings, pensions, annuities, Social Security retirement benefits, and any other income in the calendar year prior to the interview. Both household wealth and income were adjusted for household size by dividing them by the square root of the number of individuals in the household. For missing values of wealth and income, we used the imputed values provided by Rand [48]. We created three categories based on quartiles of household wealth or income in each wave to reflect one’s relative rank within the population: 1 (below 25%), 2 (25%-75%), and 3 (above 75%). Wealth was a particularly important indicator of the financial situation in the population we examined because they were beginning to transition to retirement when they depended much more on accumulated assets than income.

Psychosocial factors included current marital status, number of marriages, number of living children, religious preference, and the Center for Epidemiologic Studies Depression (CESD). CESD was a summary score of eight measures: felt depressed, everything was an effort, sleep was restless, was happy, felt lonely, sad, could not get going, and enjoyed life. Each measure was a yes/no indicator of the respondent’s feelings much of the time over the week prior to the interview. CESD score ranged from 0 to 8.

Physical status included activities of daily living (ADL) that the subject could not do or had difficulty doing, instrumental activities of daily living (IADL) that the subject could not do or had difficulty doing, and the number of chronic diseases. ADL included bathing, eating, dressing, walking across a room, and getting in or out of bed. IADL included using a telephone, taking medication, handling money, shopping, and preparing meals. The number of chronic diseases was a summary score of ever having psychiatric problems (emotional, nervous, or psychiatric problems), stroke, heart disease, high blood pressure, diabetes, lung disease, cancer, and arthritis. We examined the contributions of both the number of chronic diseases and each individual disease to the CF trajectory.

Bio-behavioral factors included BMI, smoking status, and vigorous activity. BMI consisted of five categories: underweight (BMI<18.5), normal weight (18.5< = BMI<25), overweight (25< = BMI<30), obese I (30< = BMI<35), and obese II/III (BMI> = 35). Smoking status included current smoker and former smoker with never smoker as the reference group. Vigorous activity was a binary indicator for whether the respondent engaged in vigorous physical activities or a sport at least once per week.

### Statistical analysis

We used multilevel growth curve models to estimate the intercept and slope of CF over ages 54–85. CF was normally distributed with thin tails, which met the assumption of the growth models. Multilevel growth models can easily consider the heterogeneity in individual developmental trajectories, including age- and non-age-related slope of change, estimate the relative contribution of each factor to the variation in both the level and slope of CF, accommodate unbalanced panel design, and are generally more efficient than fixed effect models [49]. All models were random-intercept and random-slope models with an unstructured variance-covariance and were estimated using maximum likelihood. The unconditional mean model setup was specified as:

$$Y_{it}=\beta_{0}+\vartheta_{0i}+\varepsilon_{it},$$

$$\vartheta_{0i}∼N(0,\sigma_{0}^{2}),\varepsilon_{it}∼N(0,\sigma_{\epsilon }^{2})$$

where *Y*_it_ was individual i’s CF at age t, *β*_0_ was the grand mean across individuals and times, *ϑ*_0*i*_ was a random intercept (i.e., person-specific means), and ***ε***_***it***_ was the error term (i.e., within-person deviations). Variance components yielded information about between-person residual variance in intercept $\sigma_{0}^{2}$ and within-person residual variance $\sigma_{\epsilon }^{2}$. Intraclass correlation ($\rho =\frac{\sigma_{0}^{2}}{\sigma_{0}^{2}+\sigma_{\epsilon }^{2}}$) compared the relative magnitude of these variance components by estimating the proportion of total variation in CF that lied “between” individuals. Adding age to this model, we got the unconditional growth model:

$$Y_{it}=\beta_{0}+\beta_{1}{Age}_{it}+\beta_{2}{AgeSq}_{it}+\vartheta_{0i}+\vartheta_{1i}{Age}_{it}+\varepsilon_{it},$$

$$\vartheta_{0i}∼N(0,\sigma_{0}^{2}),\vartheta_{1i}∼N(0,\sigma_{1}^{2}),\varepsilon_{it}∼N(0,\sigma_{\epsilon }^{2})$$

*Age*_*it*_ was the time metric. A quadratic function of age was included to model the possible accelerating declining pattern of CF in older ages. Age was centered at 54 so that the intercept *β*_0_ indicated the level of CF at initial age 54, and the slope *β*_1_ and *β*_2_ referred to the trajectory after age 54. *ϑ*_1*i*_ was a random slope. Variance component $\sigma_{0}^{2}$ indicated the between-person residual variance in initial status (age 54); $\sigma_{1}^{2}$ indicated the between-person residual variance in the rate of change (i.e., age slope); and $\sigma_{\epsilon }^{2}$ indicated the within-person residual variance. Proportional reduction in $\sigma_{\epsilon }^{2}$ from unconditional mean model to unconditional growth model indicated the percentage of within-person variation in CF associated with age. The unexplained was the within-person non-age-related variation in CF. We focused on explaining the between-person residual variance in intercept ($\sigma_{0}^{2}$) and slope ($\sigma_{1}^{2}$) by adding time-fixed early life conditions and baseline adulthood factors and their interactions with age to the unconditional growth model. Proportional reduction in variance components $\sigma_{0}^{2}$ and slope $\sigma_{1}^{2}$ indicated the percentage of between-person variation in CF at age 54 and the age slope of CF after age 54 associated with the predictors. We further examined the contribution of time-varying adulthood factors (socioeconomic, psychosocial, physical, and bio-behavioral) to within-person residual variance $\sigma_{\epsilon }^{2}$ by adding them to the unconditional growth model.

In the preceding steps, we focused on disentangling the contributions of risk and protective factors to the intercept and slope (age- and non-age-related) of CF. We further explored how CF trajectories might depend on baseline cognition and dementia status. We re-estimated the unconditional growth model removing the first observation (i.e., baseline cognition) for each individual, and then adding baseline CF or dementia status and their interactions with age to determine how much they might explain the new intercept and slope of CF. These analyses revealed how much CF trajectories might differ by individuals with different levels of cognition and different statuses of cognition (normal, CIND, demented). We used SAS proc mixed package to estimate multilevel growth models of CF trajectory over ages. Supporting Information 2 in S1 File provides the syntax for these statistical analyses.

## Results

**Fig 1:**
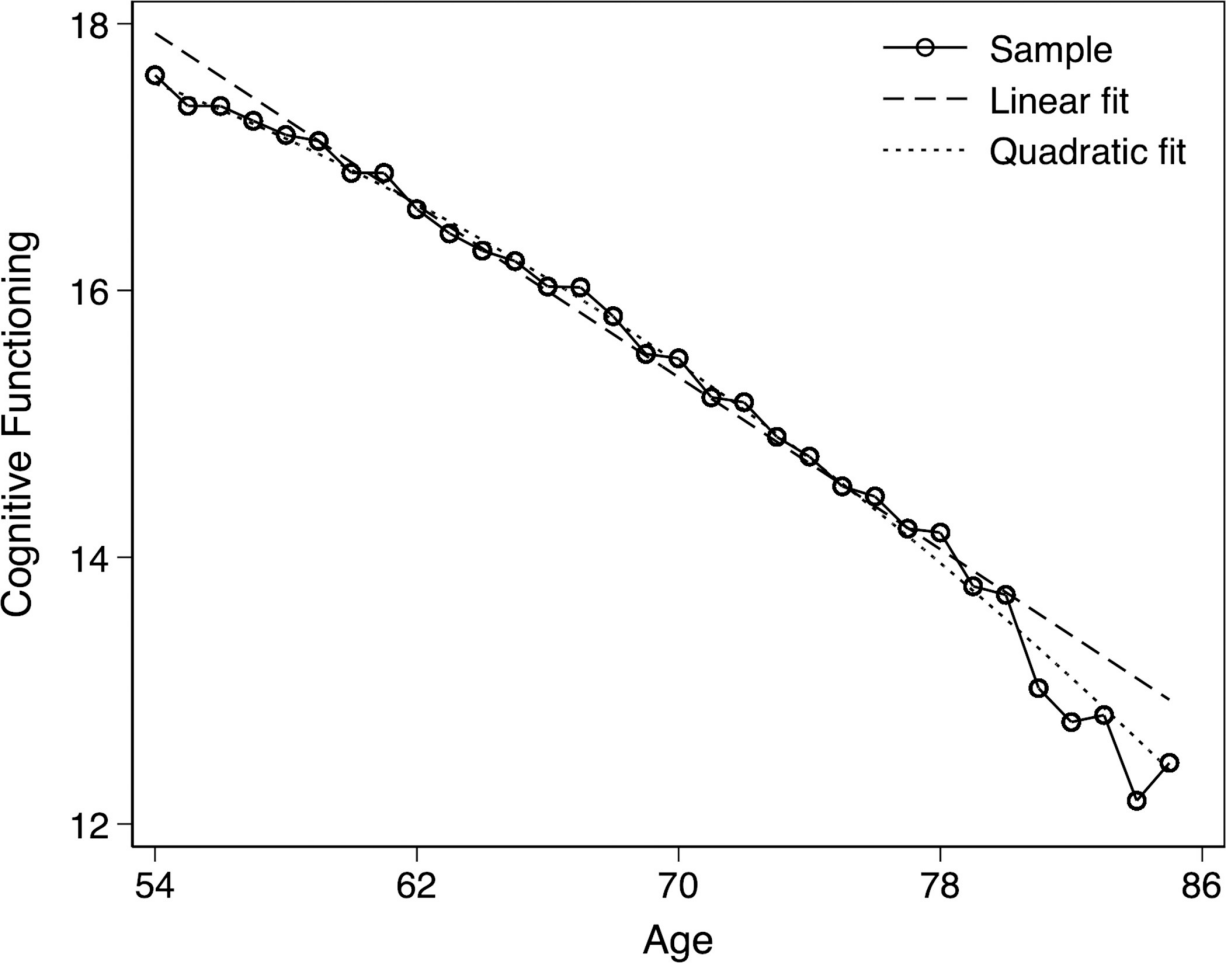
Means of cognitive functioning over ages at cohort level.

Fig 1 shows the empirical pattern and two different model fits of CF trajectory in ages 54–85. The linear model fit did not accurately capture the trajectory before age 60 and after age 80. Instead, a quadratic function of age aligned very closely with the empirical pattern. We further estimated multilevel growth models with a linear function of age and a quadratic function of age and found Bayesian information criterion (BIC) for the linear model was 280,417, bigger than 280,179 for the quadratic model. Therefore, we used a quadratic function of age to model the age slope of the CF trajectory in this sample. We also tried a polynomial with higher levels (e.g., cubic), which, however, was not significant and did not improve the model fit.

### Between-person variation in intercept and age slope of CF

Table 2 presents the fixed effect estimates and variance component from 33 multilevel linear growth models, where both linear and quadratic function of age were included. To simplify the presentation, the estimates of the quadratic function of age were not reported, and statistically non-significant coefficients were omitted from the table. Supporting Information 3 in S1 File shows all the coefficient estimates. Model (1) is the unconditional mean model. Intraclass correlation ($\rho =\frac{\sigma_{0}^{2}}{\sigma_{0}^{2}+\sigma_{\epsilon }^{2}}$) was 0.55, which meant an estimated 55% of the total variation in CF was attributable to differences between individuals. Model (2) is the unconditional growth model. Age was centered at age 54 so the intercept *β*_0_ indicated that the level of CF at the initial age of 54 was 17.383. Between-person residual variance in intercept was 13.301 and between-person residual variance in slope was 0.0179. It seems there was much more variation in intercept than slope. Since their means were different, we constructed coefficients of relative variation by dividing residual variance by their corresponding mean, which was 0.765 (= 13.301/17.383) for intercept and 0.263 (= 0.0179/0.068) for slope. So, the variation in intercept was about 3 times as large as the variation in slope. Within-person residual variance decreased from 8.946 in Model (1) to 6.866 in Model (2), which indicated that age explained 23% of within-person variation in CF. The unexplained was the non-age-related within-person change in CF.

**Table 2:** Baseline predictors of intercepts and slopes of change of cognitive functioning from multilevel linear growth models, health retirement study, 1996–2016.

Model^a^		Fixed effects		Between-person residual variance in intercept $\sigma_{0}^{2}$	Between-person residual variance in slope $\sigma_{1}^{2}$	Within-person residual variance $\sigma_{\epsilon }^{2}$	BIC
		Intercept	Slope^b^				
(1)	Unconditional mean model	15.520 (0.042)***		10.903 (0.212)***		8.946 (0.058)***	289334
(2)	Unconditional growth model	17.383 (0.070)***	-0.068 (0.008)***	13.301 (0.346)***	0.0179 (0.001)***	6.866 (0.048)***	280179
**Demographic Characteristics**							
(3)	Men	-1.145 (0.106)***	0.024 (0.005)***	13.034 (0.340)***	0.0178 (0.001)***	6.865 (0.048)***	280076
(4)	Race (ref = White)			11.697 (0.317)***	0.0177 (0.001)***	6.870 (0.048)***	279064
	Black	-2.932 (0.144)***	-0.033 (0.008)***				
	Hispanic	-3.097 (0.187)***					
	Others	-1.484 (0.366)***					
**Childhood Conditions**							
(5)	Childhood health (ref = excellent)			13.040 (0.342)***	0.0179 (0.001)***	6.866 (0.048)***	280035
	Very good	-0.468 (0.129)***					
	Above average	-1.343 (0.146)***					
	Fair	-0.905 (0.250)***					
	Poor	-1.770 (0.456)***					
(6)	Adult height		0.070 (0.027)**	13.309 (0.346)***	0.0179 (0.001)***	6.866 (0.048)***	280176
(7)	Childhood finance (ref = pretty well off)			13.121 (0.343)***	0.0179 (0.001)***	6.867 (0.048)***	280093
	About average	-0.865 (0.230)***					
	Poor	-1.605 (0.240)***					
(8)	Parental education	0.321 (0.014)***		11.895 (0.322)***	0.0178 (0.001)***	6.872 (0.048)***	279377
**Adult Socioeconomic Status**							
(9)	Years of education	0.601 (0.016)***		9.944 (0.285)***	0.0176 (0.001)***	6.876 (0.048)***	277955
(10)	College degree	2.542 (0.128)***	0.020 (0.006**	12.231 (0.335)***	0.0177 (0.001)***	6.872 (0.048)***	279430
(11)	Occupation (ref = Blue collar)	2.272 (0.108)***	0.018 (0.006**	12.116 (0.325)***	0.0177 (0.001)***	6.869 (0.048)***	279347
(12)	Household income (ref = bottom 25%)			11.791 (0.318)***	0.0176 (0.001)***	6.863 (0.048)***	279208
	25%-75%	2.269 (0.152)***					
	75%+	3.623 (0.158)***	0.019 (0.009)*				
(13)	Household wealth (ref = bottom 25%)			11.818 (0.319)***	0.0178 (0.001)***	6.863 (0.048)***	279227
	25%-75%	2.086 (0.131)***					
	75%+	3.405 (0.147)***	0.019 (0.008)*				
**Adult Psychosocial Factors**							
(14)	Marital status (ref = married)			13.223 (0.344)***	0.0178 (0.001)***	6.866 (0.048)***	280131
	Partnered	-0.678 (0.296)*					
	Separated/divorced	-0.561 (0.156)***					
	Widowed	-0.623 (0.199)**	-0.022 (0.015)*				
	Never married	-0.584 (0.299)*					
(15)	Number of marriages (ref = 1)			13.294 (0.346)***	0.0179 (0.001)***	6.867 (0.048)***	280187
	0	-0.569 (0.290)*					
	2						
	3						
	4+						
(16)	Number of living children	-0.193 (0.026)***		13.154 (0.343)***	0.0180 (0.001)***	6.866 (0.048)***	280107
(17)	Religion (ref = none)			13.275 (0.346)***	0.0178 (0.001)***	6.867 (0.048)***	280134
	Protestant		-0.031 (0.014)*				
	Catholic						
	Jewish						
	Other		-0.091 (0.036)*				
(18)	CESD	-0.500 (0.029)***	-0.004 (0.002)*	12.416 (0.331)***	0.0179 (0.001)***	6.867 (0.048)***	279635
**Adult Bio-behaviors and Diseases**							
(19)	BMI category (ref = normal weight)			13.232 (0.345)***	0.0179 (0.001)***	6.867 (0.048)***	280120
	Underweight						
	Overweight	-0.455 (0.126)***					
	Class I obese	-0.616 (0.155)***					
	Class II/III obese	-0.922 (0.218)***	-0.034 (0.011)**				
(20)	Smoking status (ref = never smoker)			13.230 (0.344)***	0.0179 (0.001)***	6.866 (0.048)***	280128
	Former smoker	-0.256 (0.120)*					
	Current smoker	-0.431 (0.143)**	-0.024 (0.008)**				
(21)	Vigorous activity	0.420 (0.107)***		13.264 (0.345)***	0.0179 (0.001)***	6.866 (0.048)***	280166
(22)	Number of chronic diseases	-0.490 (0.045)***	-0.010 (0.002)***	12.936 (0.338)***	0.0179 (0.001)***	6.863 (0.048)***	279883
(23)	High blood pressure	-0.895 (0.110)***		13.124 (0.342)***	0.0179 (0.001)***	6.865 (0.048)***	280063
(24)	Diabetes	-1.275 (0.174)***	-0.040 (0.010)***	13.101 (0.342)***	0.0178 (0.001)***	6.864 (0.048)***	279998
(25)	Lung disease	-0.744 (0.249)**		13.278 (0.345)***	0.0179 (0.001)***	6.866 (0.048)***	280174
(26)	Heart disease	-0.475 (0.165)**	-0.017 (0.009)*	13.271 (0.345)***	0.0179 (0.001)***	6.865 (0.048)***	280156
(27)	Stroke	-2.144 (0.321)***		13.153 (0.343)***	0.0179 (0.001)***	6.865 (0.048)***	280075
(28)	Psychiatric problems	-1.537 (0.202)***	-0.025 (0.011)*	13.125 (0.342)***	0.0179 (0.001)***	6.865 (0.048)***	280051
(29)	Cancer	0.635 (0.221)**		13.284 (0.346)***	0.0179 (0.001)***	6.866 (0.048)***	280178
(30)	Arthritis	-0.584 (0.108)***		13.218 (0.344)***	0.0179 (0.001)***	6.867 (0.048)***	280121
(31)	ADL	-0.963 (0.086)***	-0.011 (0.005)*	12.857 (0.338)***	0.0178 (0.001)***	6.861 (0.048)***	279921
(32)	IADL	-1.574 (0.114)***		12.665 (0.334)***	0.0178 (0.001)***	6.861 (0.048)***	279874
**All Factors**							
**(33)**	**All factors**			**8.273 (0.254)** ***	**0.0169 (0.001)** ***	**6.871 (0.048)** ***	**276537**
Sample size (number of observations): 7,068 individuals (54,400 observations)							

Note

^a^ To make the table more readable, statistically non-significant coefficients are omitted from the table.

^b^ A quadratic function of age is included in the models. Constrained by the page size, it is omitted from the table.

*p < .05

**p < .01

***p < .001

In the subsequent models, we added each baseline predictor and its interaction with age to achieve the fixed effect estimates of their relationship with the intercept and slope of CF and the random effect estimate of between-person residual variance in intercept and slope. Non-significant coefficient estimates related to either intercept or slope were not reported. We presented each predictor in one row, following the order of demographic characteristics, childhood conditions, adulthood socioeconomic status, psychosocial factors, bio-behaviors, and diseases. Men had 1.145 units less than women in CF at age 54 but had a slower rate of decline afterward than women. Non-whites had lower CF at age 54 than Whites, and Blacks had a steeper decline in CF than Whites. Better childhood conditions (including childhood health, financial situation, and parental education) predicted higher CF at age 54 but not the age slope of CF. Higher adult height (a proxy for childhood nutrition environment) was associated with a slower decline in CF after age 54.

Better adulthood socioeconomic status (including education, occupation, income, and wealth) was associated with better CF at age 54 and a slower decline afterwards, consistent with the “cumulative (dis)advantage” theory. One particularly interesting finding is years of education was not significantly associated with the age slope of CF but those with a college degree had a slower decline of CF than those without. Unmarried status was associated with worse CF and widowhood led to a steeper decline in CF than being married. A larger number of children led to a lower level of CF at age 54 but did not influence the age slope of CF. Protestant faith was associated with a steeper decline in CF than those without religious affiliations. CESD (an index of depression) predicted both a lower intercept and a steeper decline in CF.

Unhealthy bio-behaviors (morbid obesity and smoking) were linked to both a lower CF and a steeper age slope of CF decline. Vigorous activity improved the level of CF but did not alter the age slope. Chronic diseases in terms of both quantity and specificity negatively affected CF. Diabetes, heart diseases, and psychiatric problems further aggravated the decline in CF. Cancer, surprisingly, was positively associated with the level of CF. Activities limitations (ADL, IADL) decreased the level of CF and/or steepened the decline in CF.

**Fig 2:**
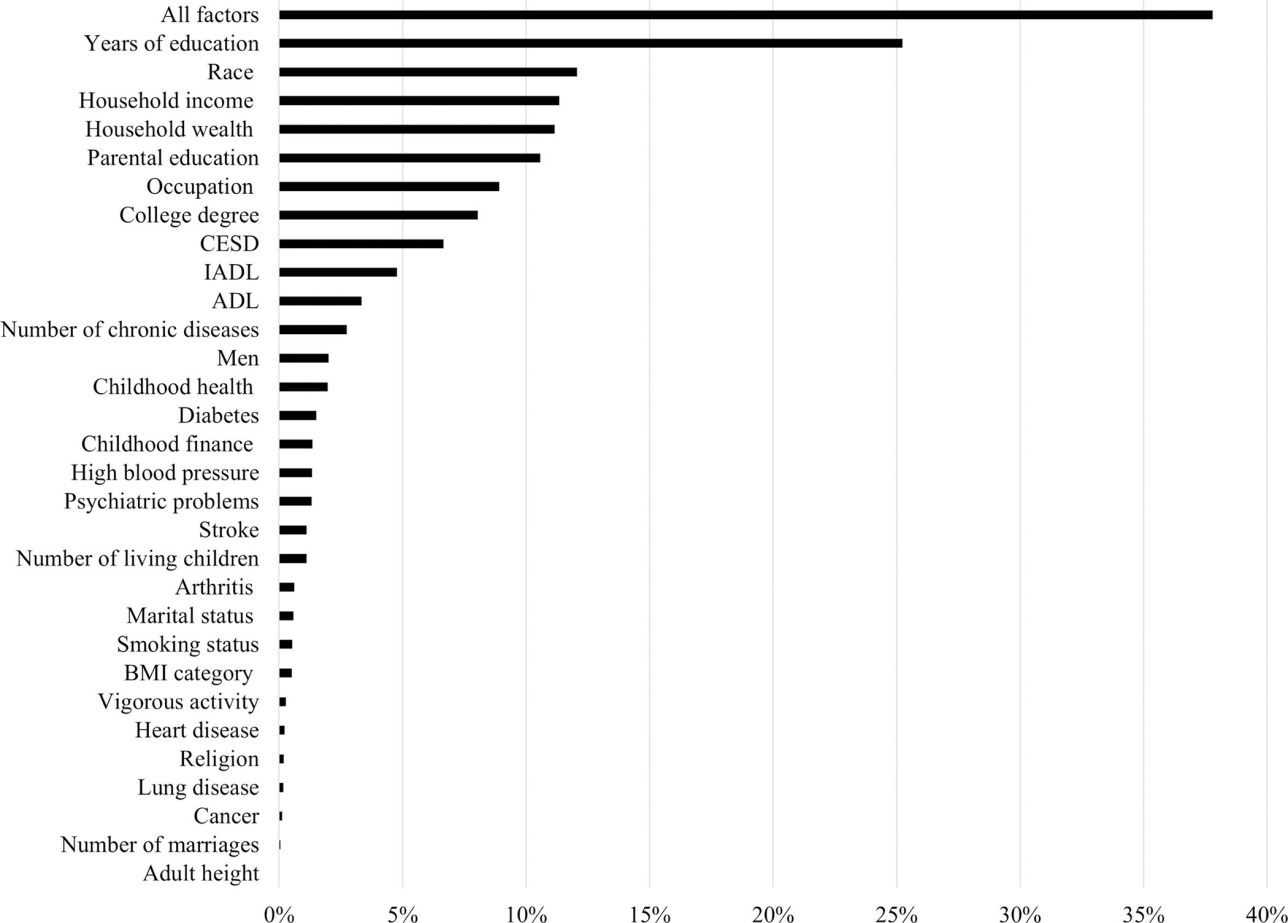
Percentage changes in between-person residual variance in intercept from unconditional growth model to subsequent models of Table 2.

**Fig 3:**
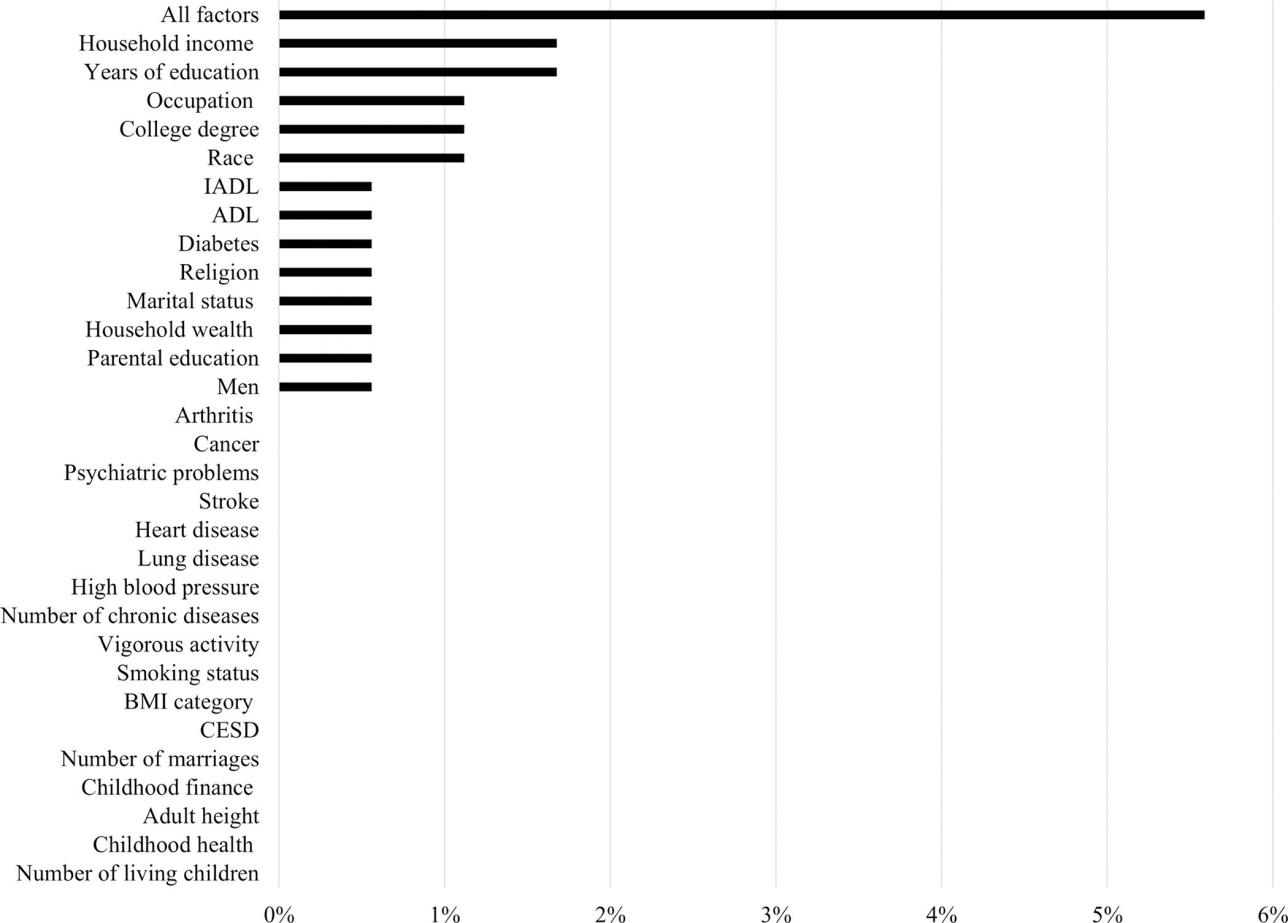
Percentage changes in between-person residual variance in slope from unconditional growth model to subsequent models of Table 2.

How much do these factors contribute to the variation in the intercept (age 54) and age slope? We calculated the percentage change in between-person residual variance in intercept and slope from unconditional growth Model (2) to subsequent models and displayed these percentage changes in Fig 2 (intercept) and Fig 3 (slope). The predictors were ordered by the magnitude of change in residual variance due to their inclusion. As Fig 2 shows, all the individual socioeconomic, psychosocial and biobehavioral factors explained 38% of the variation in intercept (age 54) (Model 33 vs. Model 2). Among these factors, education was most important (25%), followed by race, household wealth and income, parental education, occupation, and depression. The contributions of activity limitations, chronic diseases, health behaviors (obesity, smoking, vigorous activity), childhood conditions (except parental education), gender, marital status, and religion were rather small (<5%). Even though the age slope varied by some factors (e.g., adulthood socioeconomic status, health behaviors, and physical status), all these factors only explained 5.6% of the variation in slope (Model 33 vs. Model 2) as shown in Fig 3. The most important observed contributor to the intercept, education, only explained 1.7% of the variation in age slope (Model 9 vs. Model 2).

Next, we investigated whether baseline cognition at the year 1996 explained the variation in intercept and age slope of CF by re-estimating CF trajectory from 1998 to 2016 and including baseline cognition in the model. Age was centered at 56 (2 years after 1996), so the intercept *β*_0_ indicated the level of CF at age 56. As shown in Table 3, baseline dementia and CIND were associated with lower CF. But surprisingly, they were also associated with a less steep decline in CF compared to the baseline normal cognition status (Model 2). Similarly, a higher baseline cognition score was associated with a steeper age slope of CF (Model 3). Baseline cognition explained 54% of the variation in intercept (age 56) (Model 3 vs. Model 1) but only 4% of the variation in age slope (Model 3 vs. Model 1).

**Table 3:** Parameter estimates from multilevel linear growth models of cognitive functioning trajectory on baseline cognition score and dementia status, health retirement study, 1998–2016.

Model		Fixed effects			Between-person residual variance in intercept $\sigma_{0}^{2}$	Between-person residual variance in slope $\sigma_{1}^{2}$	Within-person residual variance $\sigma_{\epsilon }^{2}$	BIC
		Intercept	Linear slope	Quadratic slope				
(1)	Unconditional growth model	17.433 (0.074)***	-0.102 (0.009)***	-0.004 (0.000)***	13.334 (0.371)***	0.0208 (0.001)***	6.748 (0.051)***	244125
(2)	Baseline dementia (ref = normal)				9.494 (0.295)***	0.0205 (0.001)***	6.750 (0.051)***	241908
	CIND	-5.635 (0.176)***	0.022 (0.011)*					
	Dementia	-9.814 (0.462)***	0.091 (0.029)**					
(3)	Baseline cognition	0.632 (0.011)***	-0.004 (0.001)***		6.154 (0.233)***	0.0200 (0.001)***	6.766 (0.051)***	239646
Sample size (number of observations): 6,822 individuals (47,332 observations)								

Note

*p < .05

**p < .01

***p < .001

### Within-person non-age-related variability of CF

In order to test what contributed to the within-person non-age-related variability of CF, we added time-varying predictors to the unconditional growth model. These predictors included time-varying adulthood factors (socioeconomic, psychosocial, physical, and bio-behavioral) and excluded gender, race, time-constant early life, and adulthood conditions (e.g., education). Table 4 shows the within-person residual variance from 22 models. Model 1 was the unconditional growth model, the same model as Model 2 in Table 2. Since age and age squared were included in the model, the within-personal residual variance indicated the non-age-related change in CF. The subsequent models added the aforementioned time-varying predictors. But adding them had little impact on the within-person residual variance. In other words, these factors did not really explain the non-age-related variability in CF within individuals.

**Table 4:** Time-varying predictors of within-person change of cognitive functioning from multilevel linear growth models, health retirement study, 1996–2016.

Model		Within-person residual variance $\sigma_{\epsilon }^{2}$	BIC
(1)	Unconditional growth model	6.866 (0.048)***	280179
(2)	Household income	6.892 (0.048)***	279894
(3)	Household wealth	6.902 (0.048)***	279760
(4)	Marital status	6.864 (0.048)***	280159
(5)	Number of marriages	6.861 (0.048)***	280172
(6)	Number of living children	6.865 (0.048)***	280140
(7)	CESD	6.873 (0.048)***	279945
(8)	BMI category	6.860 (0.048)***	280173
(9)	Smoking status	6.862 (0.048)***	280160
(10)	Vigorous activity	6.865 (0.048)***	280148
(11)	Number of chronic diseases	6.865 (0.048)***	279973
(12)	High blood pressure	6.863 (0.048)***	280129
(13)	Diabetes	6.869 (0.048)***	280135
(14)	Lung disease	6.863 (0.048)***	280175
(15)	Heart disease	6.860 (0.048)***	280152
(16)	Stroke	6.861 (0.048)***	280003
(17)	Psychiatric problems	6.867 (0.048)***	280060
(18)	Cancer	6.862 (0.048)***	280182
(19)	Arthritis	6.862 (0.048)***	280173
(20)	ADL	6.864 (0.048)***	279884
(21)	IADL	6.859 (0.048)***	279568
**(22)**	**All factors**	**6.900 (0.048)** ***	**278713**
Sample size (number of observations): 7,068 individuals (54,400 observations)			

Note

***p < .001

## Discussion

This study models cognitive trajectories in individuals ages 54–85 and investigates the contribution of a wide range of factors over the life course to CF trajectories using HRS data from the years 1996–2016. We investigated not only the level and age-related slope of CF like many prior studies, but also the non-age-related slope of change seldom examined before. We found that the population was more dissimilar in the level rather than the age slope of CF. The variation in level was about three times as large as the variation in age slope. In addition, age only explained 23% of the within-person variation in CF from age 54 to 85. The remaining 77% of the within-person variation could not be explained by age.

We further found that all the observed factors accounted for 38% of the variation in the level of CF at age 54, among which individual socioeconomic status (e.g., education, income, wealth, occupation, race, parental education) mattered most, while early life conditions (except parental education), health behaviors (obesity, smoking, vigorous activity), activity limitations, and diseases did not make much of a contribution. This does not mean that they were not statistically significantly associated with the level of CF. In fact, many of them were. It means that their contributions to the variation in the level of CF at the population level were rather small. These findings point to the predominant importance of socioeconomic conditions in shaping the level of CF.

CF followed an accelerating decline in older ages at the cohort level. The age slope did not vary by most early life conditions, which is consistent with some prior studies [13, 39, 40, 43, 44] but incongruent with the “cumulative (dis)advantage” theory. However, as explained earlier, most early life conditions (except parental education) were not important contributors to the level of CF in later life either. The age-related change of CF did vary by some adulthood socioeconomic, psychosocial, and health factors in a direction expected by the “cumulative (dis)advantage” theory. For example, the slope of decline in CF was less steep among individuals with higher education, income, and wealth, but steeper among individuals with depression, unhealthy behaviors (smoking, obesity), cardiovascular diseases risk factors, and activity limitations. The findings regarding depression, smoking, and physical health are consistent with prior studies [15–18, 25]. The findings regarding socioeconomic status, however, are inconsistent with some prior studies [41, 42]. Those studies might be unable to identify the age-varying effects of these predictors due to a narrow age cohort or small sample size [13].

One particularly interesting finding is that education (in years) was not associated with the age slope of CF, but a college degree was. The null finding for years of education is consistent with some studies [40, 50], but the significant finding for college degree supports the “cognitive reserve” hypothesis [38], which claims that cognitive reserve capacity may delay the manifestation of neuropathology that occurs as a result of aging-related or pathologic processes [37]. These discrepancies emphasize the importance of differentiation between years of education and education degrees. Prior studies found that the relationship between education and health was not linear. Instead, there was a step-change reduction in mortality risk upon attainment of a high school diploma, at which point mortality risk continued declining linearly with years of higher education but at a faster rate [51]. This pattern was consistent with a less steep decline in CF associated with college degree found in our study. College may provide an especially rich environment for cognitive development beyond pre-college education. College education also increases one’s life expectations and incentives to engage in healthier behaviors, leads to occupations with more mental stimulations, and is very consequential for one’s income and higher quality of social connections who have greater formal education [52–54], all of which can contribute to a slower decline of CF in later life.

Despite the statistically significant association with age slope, all the controlled factors only explained 5.6% of the variation in age slope at the population level. Education, the most important observed contributor to the level of CF, only explained 1.7% of the variation in age slope. Therefore, the majority of variation in age slope was not explained. Prior studies emphasize the importance of education in shaping the period trend in the dementia prevalence [55, 56]. It merits further investigation on whether education explains the age-related progression of dementia over time. Moreover, we found all the observed individual socioeconomic indicators, bio-behaviors, and physical health measures could not explain the within-person non-age-related variability in CF from age 54 to 85.

These findings raise the importance of distinguishing between statistical significance and substantive contribution. Although many factors are statistically associated with the level and slope of CF, their contributions to the population variation, especially the slope, are rather small. Future research is urgently needed to discover the main determinants of the slope of decline to slow down the progression of cognitive impairment and dementia. Unfortunately, the determinants examined in this study have a very minor impact on either the age slope or non-age-related change of CF.

We did supplemental analyses to assess the sensitivity of the results to the exclusion of proxy respondents by examining the trajectory in cognitive limitation (CL), a measure developed by Langa and Weir [46]. In the Langa-Weir specification, a total CF score of 0–6 points was labeled as “demented,” 7–11 as “CIND,” and 12–27 as “normal.” We created an indicator “cognitive limitation” by assigning 1 if the total score was 0–11, and 0 otherwise [57]. CL among sample persons with proxies was based on the sum of three variables: proxy’s assessment of memory ranging from excellent (0) to poor (4; range 0–4), number of five IADLs that a sample subject could not do or had difficulty doing (score 0–5), and the interviewer’s assessment of difficulty in completing the interview because of the subject’s CLs (score of 0 = none, 1 = some, and 2 = prevents completion). A summary score of 0–2 was classified as normal cognition, and 3–11 as CL [46]. Overall, the findings were not qualitatively different from those based on CF.

There are limitations in our analysis. First, we used the HRS cognitive test score as a measurement of cognitive functioning. We found that a higher baseline cognition was associated with a steeper decline in CF (Table 3, Model 2 and 3), which was probably due to the floor effect. For those with low baseline CF or dementia, there is not much room to obtain a lower cognition test score, and this may have caused the rate of decline to be smaller for these individuals compared to those with a high baseline CF or without dementia. But this may also reflect the “law of initial value,” whereby those with initially higher abilities decline faster as there is more room for them to do so [58]. In other words, this phenomenon is not a result of the specific CF measure we used, but due to the floor effect of the underlying level of cognition. Nonetheless, it is important to replicate the analysis with clinical and neuropsychiatric assessments of cognitive function. Second, even though HRS cognitive battery has been widely used in many studies [55, 57, 59, 60], it has been mainly focused on memory function and lacks other cognitive domains (e.g., processing speed and executive function). Even though the validity of this cognitive battery has been verified by a clinically evaluated neuropsychological examination in the Aging Demographics and Memory Study [46], it is important to replicate the analyses on other cognitive domains when data become available. Third, even though we examined many possible determinants of CF, we missed some core factors, e.g., APOE e4 genotype. However, our study probably included more comprehensive potential predictors than previous studies. Future studies can build on our approach to investigate the contribution of other possible determinants to both the level and slope (age- and non-age-related) of CF.

Fourth, even though at the cohort level, the decline in CF clearly follows a linear and quadratic curve with aging, there are substantial variations at the individual level as indicated by the between-person residual variation in age slope and within-person residual variance. Our analysis has not successfully identified the causes of these discrepancies. Future studies should ascertain explanations for these discrepancies, which is the key to understanding the causes of age and non-age-related slope of cognitive decline. Fifth, since this study is based on observational data, we should be cautious about the causal interpretation of the findings. Our goal was not to establish causal links between the CF trajectories. Instead, we intended to identify important correlates and their substantive contributions. Nonetheless, future studies should use a more rigorous research design to estimate the causal effect of the identified correlates. Carefully designed interventional studies may identify the causal effect of certain variables (e.g., behavioral factors) in a short follow-up period, but many sociodemographic and psychosocial variables cannot be randomly assigned, and a long follow-up period may also suffer from non-random attrition. Therefore, observational studies like this study still provide important insights regarding the correlation among factors.

Sixth, this study does not address the likely dynamic interactions among the predictors, which is far beyond the scope of this paper. Our goal is just to estimate both the gross and net relationships between these predictors and CF trajectories, accounting for other correlated factors. Seventh, like all the longitudinal data, HRS may suffer from attrition bias, due to either mortality or non-mortality dropout. We conducted two analyses to test the attrition bias. First, we tested whether the attrition in the next wave was associated with the cognition score in the previous wave (conditional on the observed covariates) and found the missing mechanism follows MAR (missing at random). Second, we applied the shared parameter model of Follmann and Wu [61] to account for the mortality selection and non-mortality dropout biases jointly, and the major patterns were sustained, which may be because the attrition was MAR-conditional on the observables. Notwithstanding, attrition is a pervasive problem in aging research and should warrant more attention in future research.

Despite these limitations, this paper utilizes large nationally representative longitudinal data with a long follow-up period to investigate the relative contribution of a broad range of possible predictors to both the level and slope of CF trajectories in older ages. Due to its large sample size and broad age cohort, it provides a more robust analysis than prior studies [13]. We not only addressed the contribution of baseline predictors to variation in the level and age slope of CF but also tested the contribution of time-varying predictors to the variation in non-age-related change in CF, an endeavor not undertaken in prior studies to our knowledge. We found adulthood socioeconomic conditions played a more prominent role in shaping the level of CF than early life conditions and adulthood health behaviors and diseases. However, all these observed factors did not explain much of the variation in CF progression over ages at the population level even though many of them had a statistically significant association with it. These findings point to the importance of future research to continue discovering the essential predictors of the rate of cognitive decline in older ages.

## Supporting information

S1 File (DOCX)

## References

[1] Manton KC, Gu XL, Ukraintseva SV. Declining prevalence of dementia in the U.S. elderly population. Adv Gerontol. 2005;16: 30–37. 16075674

[2] Prince MJ, Wimo A, Guerchet MM, Ali GC, Wu Y-T, Prina M. World Alzheimer Report 2015-The global impact of dementia: An analysis of prevalence, incidence, cost and trends. 2015. Available: http://kclpure.kcl.ac.uk/portal/en/publications/world-alzheimer-report-2015—the-global-impact-of-dementia(ae525fda-1938-4892-8daa-a2222a672254).html

[3] Alzheimer’s Association. Costs of Alzheimer’s to Medicare and Medicaid. 2020 [cited 26 Aug 2020]. Available: https://act.alz.org/site/DocServer/2012_Costs_Fact_Sheet_version_2.pdf?docID=7161

[4] Boyle PA, Wilson RS, Yu L, Barr AM, Honer WG, Schneider JA, et al. Much of late life cognitive decline is not due to common neurodegenerative pathologies. Ann Neurol. 2013;74: 478–489. doi: 10.1002/ana.23964 23798485 PMC3845973

[5] Zaninotto P, David Batty G, Allerhand M, Deary IJ. Cognitive function trajectories and their determinants in older people: 8 years of follow-up in the English Longitudinal Study of Ageing. J Epidemiol Community Health. 2018;0: 1–10. doi: 10.1136/jech-2017-210116 29691286 PMC6204948

[6] Batty GD, Deary IJ, Zaninotto P. Association of cognitive function with cause-specific mortality in middle and older age: Follow-up of participants in the English Longitudinal Study of Ageing. Am J Epidemiol. 2016;183: 183–190. doi: 10.1093/aje/kwv139 26803665 PMC4724091

[7] Plassman BL, Williams JW, Burke JR, Holsinger T, Benjamin S. Systematic review: Factors associated with risk for and possible prevention of cognitive decline in later life. Annals of Internal Medicine. American College of Physicians; 2010. pp. 182–193. doi: 10.7326/0003-4819-153-3-201008030-00258 20547887

[8] Dannefer D. Cumulative advantage/disadvantage and the life course: cross-fertilizing age and social science theory. J Gerontol B Psychol Sci Soc Sci. 2003;58: S327–37. doi: 10.1093/geronb/58.6.s327 14614120

[9] Hatch SL. Conceptualizing and identifying cumulative adversity and protective resources: implications for understanding health inequalities. J Gerontol B Psychol Sci Soc Sci. 2005;60 Spec No 2: 130–134. doi: 10.1093/geronb/60.special_issue_2.s130 16251584

[10] O’Rand Henretta. Age and inequality: Diverse pathways through later life. Denver, CO: Westview. 1999.

[11] Cherbuin N, Reglade-Meslin C, Kumar R, Jacomb P, Easteal S, Christensen H, et al. Risk factors of transition from normal cognition to mild cognitive disorder: The PATH through life study. Dement Geriatr Cogn Disord. 2009;28: 47–55. doi: 10.1159/000229025 19628940

[12] Salthouse TA. Correlates of cognitive change. J Exp Psychol Gen. 2014;143: 1026–1048. doi: 10.1037/a0034847 24219021 PMC4017000

[13] Ritchie SJ, Tucker-Drob EM, Cox SR, Corley J, Dykiert D, Redmond P, et al. Predictors of ageing-related decline across multiple cognitive functions. Intelligence. 2016;59: 115–126. doi: 10.1016/j.intell.2016.08.007 27932854 PMC5127886

[14] Yaffe K, Fiocco AJ, Lindquist K, Vittinghoff E, Simonsick EM, Newman AB, et al. Predictors of maintaining cognitive function in older adults: the Health ABC study. Neurology. 2009;72: 2029–2035. doi: 10.1212/WNL.0b013e3181a92c36 19506226 PMC2692177

[15] Anstey KJ, Von Sanden C, Salim A, O’Kearney R. Smoking as a risk factor for dementia and cognitive decline: A meta-analysis of prospective studies. Am J Epidemiol. 2007;166: 367–378. doi: 10.1093/aje/kwm116 17573335

[16] Emery CF, Finkel D, Pedersen NL. Pulmonary function as a cause of cognitive aging. Psychol Sci. 2012;23: 1024–1032. doi: 10.1177/0956797612439422 22864997 PMC3752604

[17] Gale CR, Allerhand M, Deary IJ. Is there a bidirectional relationship between depressive symptoms and cognitive ability in older people? A prospective study using the English Longitudinal Study of Ageing. Psychol Med. 2012;42: 2057–2069. doi: 10.1017/S0033291712000402 23206378 PMC3435872

[18] Yaffe K, Blackwell T, Gore R, Sands L, Reus V, Browner WS. Depressive symptoms and cognitive decline in nondemented elderly women: a prospective study. Arch Gen Psychiatry. 1999;56: 425–430. doi: 10.1001/archpsyc.56.5.425 10232297

[19] Wilson RS, Schneider JA, Boyle PA, Arnold SE, Tang Y, Bennett DA. Chronic distress and incidence of mild cognitive impairment. Neurology. 2007;68: 2085–2092. doi: 10.1212/01.wnl.0000264930.97061.82 17562829

[20] Knopman DS, Mosley TH, Catellier DJ, Coker LH. Fourteen-year longitudinal study of vascular risk factors, APOE genotype, and cognition: the ARIC MRI Study. Alzheimers Dement. 2009;5: 207–214. doi: 10.1016/j.jalz.2009.01.027 19362884

[21] Yaffe K, Kanaya A, Lindquist K, Simonsick EM, Harris T, Shorr RI, et al. The metabolic syndrome, inflammation, and risk of cognitive decline. JAMA. 2004;292: 2237–2242. doi: 10.1001/jama.292.18.2237 15536110

[22] Blair CK, Folsom AR, Knopman DS, Bray MS, Mosley TH, Boerwinkle E. APOE genotype and cognitive decline in a middle-aged cohort. Neurology. 2005;64: 268–276. doi: 10.1212/01.WNL.0000149643.91367.8A 15668424

[23] Carmelli D, Swan GE, Reed T, Miller B, Wolf PA, Jarvik GP, et al. Midlife cardiovascular risk factors, ApoE, and cognitive decline in elderly male twins. Neurology. 1998;50: 1580–1585. doi: 10.1212/wnl.50.6.1580 9633697

[24] Yaffe K, Barnes D, Nevitt M, Lui LY, Covinsky K. A prospective study of physical activity and cognitive decline in elderly women: women who walk. Arch Intern Med. 2001;161: 1703–1708. doi: 10.1001/archinte.161.14.1703 11485502

[25] Finkel D, Ernsth-Bravell M, Pedersen NL. Temporal dynamics of motor functioning and cognitive aging. Journals of Gerontology—Series A Biological Sciences and Medical Sciences. 2016;71: 109–116. doi: 10.1093/gerona/glv110 26286604 PMC4881822

[26] Lautenschlager NT, Cox KL, Flicker L, Foster JK, Van Bockxmeer FM, Xiao J, et al. Effect of physical activity on cognitive function in older adults at risk for Alzheimer disease: a randomized trial. JAMA. 2008;300: 1027–1037. doi: 10.1001/jama.300.9.1027 18768414

[27] Scarmeas N, Stern Y, Mayeux R, Manly JJ, Schupf N, Luchsinger JA. Mediterranean diet and mild cognitive impairment. Arch Neurol. 2009;66: 216–225. doi: 10.1001/archneurol.2008.536 19204158 PMC2653223

[28] Willis SL, Tennstedt SL, Marsiske M, Ball K, Elias J, Koepke KM, et al. Long-term effects of cognitive training on everyday functional outcomes in older adults. JAMA. 2006;296: 2805. doi: 10.1001/jama.296.23.2805 17179457 PMC2910591

[29] McCarrey AC, An Y, Kitner-Triolo MH, Ferrucci L, Resnick SM. Sex differences in cognitive trajectories in clinically normal older adults. Psychol Aging. 2016;31: 166–175. doi: 10.1037/pag0000070 26796792 PMC4783196

[30] Finkel D, Reynolds CA, McArdle JJ, Gatz M, Pedersen NL. Latent growth curve analyses of accelerating decline in cognitive abilities in late adulthood. Dev Psychol. 2003;39: 535–550. doi: 10.1037/0012-1649.39.3.535 12760521

[31] Banks J, Mazzonna F. The effect of education on old age cognitive abilities: Evidence from a regression discontinuity design. Econ J. 2012;122: 418–448. doi: 10.1111/j.1468-0297.2012.02499.x 22611283 PMC3351837

[32] Clouston SAP, Kuh D, Herd P, Elliott J, Richards M, Hofer SM. Benefits of educational attainment on adult fluid cognition: International evidence from three birth cohorts. Int J Epidemiol. 2012;41: 1729–1736. doi: 10.1093/ije/dys148 23108707 PMC3535750

[33] Koster A, Penninx BWJH, Bosma H, Kempen GIJM, Newman AB, Rubin SM, et al. Socioeconomic differences in cognitive decline and the role of biomedical factors. Ann Epidemiol. 2005;15: 564–571. doi: 10.1016/j.annepidem.2005.02.008 15922627

[34] Lyu J, Burr JA. Socioeconomic status across the life course and cognitive function among older adults: An examination of the latency, pathways, and accumulation hypotheses. J Aging Health. 2016;28: 40–67. doi: 10.1177/0898264315585504 26006338

[35] Potter GG, Plassman BL, Helms MJ, Foster SM, Edwards NW. Occupational characteristics and cognitive performance among elderly male twins. Neurology. 2006;67: 1377–1382. doi: 10.1212/01.wnl.0000240061.51215.ed 17060563

[36] Tervo S, Kivipelto M, Hänninen T, Vanhanen M, Hallikainen M, Mannermaa A, et al. Incidence and risk factors for mild cognitive impairment: a population-based three-year follow-up study of cognitively healthy elderly subjects. Dement Geriatr Cogn Disord. 2004;17: 196–203. doi: 10.1159/000076356 14739544

[37] Cagney KA, Lauderdale DS. Education, wealth, and cognitive function in later life. J Gerontol B Psychol Sci Soc Sci. 2002;57: P163–72. doi: 10.1093/geronb/57.2.p163 11867664

[38] Stern Y. What is cognitive reserve? Theory and research application of the reserve concept. J Int Neuropsychol Soc. 2002;8: 448–460. 11939702

[39] Everson-Rose SA, Mendes De Leon CF, Bienias JL, Wilson RS, Evans DA. Early life conditions and cognitive functioning in later life. Am J Epidemiol. 2003;158: 1083–1089. doi: 10.1093/aje/kwg263 14630604

[40] Tucker-Drob EM, Johnson KE, Jones RN. The cognitive reserve hypothesis: A longitudinal examination of age-associated declines in reasoning and processing speed. Dev Psychol. 2009;45: 431–446. doi: 10.1037/a0014012 19271829 PMC3230274

[41] Tyas SL, Salazar JC, Snowdon DA, Desrosiers MF, Riley KP, Mendiondo MS, et al. Transitions to mild cognitive impairments, dementia, and death: findings from the Nun Study. Am J Epidemiol. 2007;165: 1231–1238. doi: 10.1093/aje/kwm085 17431012 PMC2516202

[42] Wilson RS, Hebert LE, Scherr PA, Barnes LL, De Leon CFM, Evans DA. Educational attainment and cognitive decline in old age. Neurology. 2009;72: 460. doi: 10.1212/01.wnl.0000341782.71418.6c 19188578 PMC2677531

[43] Wilson RS, Scherr PA, Hoganson G, Bienias JL, Evans DA, Bennett DA. Early life socioeconomic status and late life risk of Alzheimer’s disease. Neuroepidemiology. 2005;25: 8–14. doi: 10.1159/000085307 15855799

[44] Zhang Z, Liu J, Li L, Xu H. The long arm of childhood in china: Early-Life conditions and cognitive function among middle-aged and older adults. J Aging Health. 2018;30: 1319–1344. doi: 10.1177/0898264317715975 28645234

[45] Fisher GG, Hassan H, Faul JD, Rodgers WL, Weir DR. Health and Retirement Study imputation of cognitive functioning measures: 1992–2014. Ann Arbor, MI: Survey Research Center University of Michigan http://hrsonline isr umich edu/modules/meta/xyear/cogimp/desc/COGIMPdd pdf Published January. 2017;13.

[46] Crimmins EM, Kim JK, Langa KM, Weir DR. Assessment of cognition using surveys and neuropsychological assessment: the Health and Retirement Study and the Aging, Demographics, and Memory Study. J Gerontol B Psychol Sci Soc Sci. 2011;66 Suppl 1: i162–i171. doi: 10.1093/geronb/gbr048 21743047 PMC3165454

[47] Case A, Paxson C. Height, health, and cognitive function at older ages. Am Econ Rev. 2008;98: 463–467. doi: 10.1257/aer.98.2.463 25152537 PMC4138508

[48] Hurd M, Meijer E, Moldoff M, Rohwedder S. Improved wealth measures in the Health and Retirement Study: Asset reconciliation and cross-wave imputation. RAND Corporation; 2016.

[49] Singer JD, Willett JB. Applied longitudinal data analysis: Modeling change and event occurrence. Oxford University Press, USA; 2003.

[50] Gottesman RF, Rawlings AM, Sharrett AR, Albert M, Alonso A, Bandeen-Roche K, et al. Impact of differential attrition on the association of education with cognitive change over 20 years of follow-up. Am J Epidemiol. 2014;179: 956–966. 24627572 10.1093/aje/kwu020 PMC3966720

[51] Montez JK, Hummer RA, Hayward MD. Educational attainment and adult mortality in the United States: A systematic analysis of functional form. Demography. 2012;49: 315–336. doi: 10.1007/s13524-011-0082-8 22246797 PMC3290920

[52] Becker GS, Mulligan CB. The endogenous determination of time preference. Q J Econ. 1997;112: 729–758.

[53] Cutler DM, Lleras-Muney A. Understanding differences in health behaviors by education. J Health Econ. 2010;29: 1–28. doi: 10.1016/j.jhealeco.2009.10.003 19963292 PMC2824018

[54] Schooler C, Mulatu MS, Oates G. The continuing effects of substantively complex work on the intellectual functioning of older workers. Psychol Aging. 1999;14: 483–506. doi: 10.1037//0882-7974.14.3.483 10509702

[55] Crimmins EM, Saito Y, Kim JK, Zhang YS, Sasson I, Hayward MD. Educational differences in the prevalence of dementia and life expectancy with dementia: Changes from 2000 to 2010. Journals of Gerontology—Series B Psychological Sciences and Social Sciences. 2018. pp. S20–S28. doi: 10.1093/geronb/gbx135 29669097 PMC6019027

[56] Hayward MD, Farina MP, Zhang YS, Kim JK, Crimmins EM. The importance of improving educational attainment for dementia prevalence trends from 2000 to 2014 among older Non-Hispanic Black and White Americans. J Gerontol B Psychol Sci Soc Sci. 2021;76: 1870–1879. doi: 10.1093/geronb/gbab015 33481025 PMC8557827

[57] Choi H, Schoeni RF, Martin LG, Langa KM. Trends in the prevalence and disparity in cognitive limitations of Americans 55–69 years old. Journals of Gerontology—Series B Psychological Sciences and Social Sciences. 2018;73: S29–S37. doi: 10.1093/geronb/gbx155 29669102 PMC6019031

[58] Wilder J. The law of initial value in neurology and psychiatry: Facts and problems. J Nerv Ment Dis. 1957;125: 73–86. doi: 10.1097/00005053-195701000-00009 13481701

[59] Hudomiet P, Hurd MD, Rohwedder S. Dementia prevalence in the United States in 2000 and 2012: Estimates based on a nationally representative study. Journals of Gerontology—Series B Psychological Sciences and Social Sciences. 2018;73: S10–S19. doi: 10.1093/geronb/gbx169 29669104 PMC6018928

[60] Zissimopoulos JM, Tysinger BC, St Clair PA, Crimmins EM. The impact of changes in population health and mortality on future prevalence of Alzheimer’s disease and other dementias in the United States. Journals of Gerontology—Series B Psychological Sciences and Social Sciences. 2018;73: S38–S47. doi: 10.1093/geronb/gbx147 29669100 PMC6019010

[61] Follmann D, Wu M. An approximate Generalized Linear Model with random effects for informative missing data. Biometrics. 1995;51: 151–168. 7766771

