# Supplementary material for: Predictors of cognitive functioning trajectories among older Americans: A new investigation covering 20 years of age- and non-age-related cognitive change
Source: PLoS One. 2023 Feb 8;18(2):e0281139. doi: 10.1371/journal.pone.0281139 (PMC9907834; doi:10.1371/journal.pone.0281139)
Supplement: S1 File — (DOCX) [file pone.0281139.s001.docx]

**Supporting Information**

Predictors of cognitive functioning trajectories among older Americans:

A new investigation covering 20 years of age- and non-age-related cognitive change

Hui Zheng^1,*^, Kathleen Cagney^2^, and Yoonyoung Choi^1^

^1^ Department of Sociology, Institute for Population Research, The Ohio State University, Columbus, Ohio, United States of America

^2^ Department of Sociology, Institute for Social Research, University of Michigan, Ann Arbor, Michigan, United States of America

**Supporting Information 1. Flowchart of the Study Sample.**

**
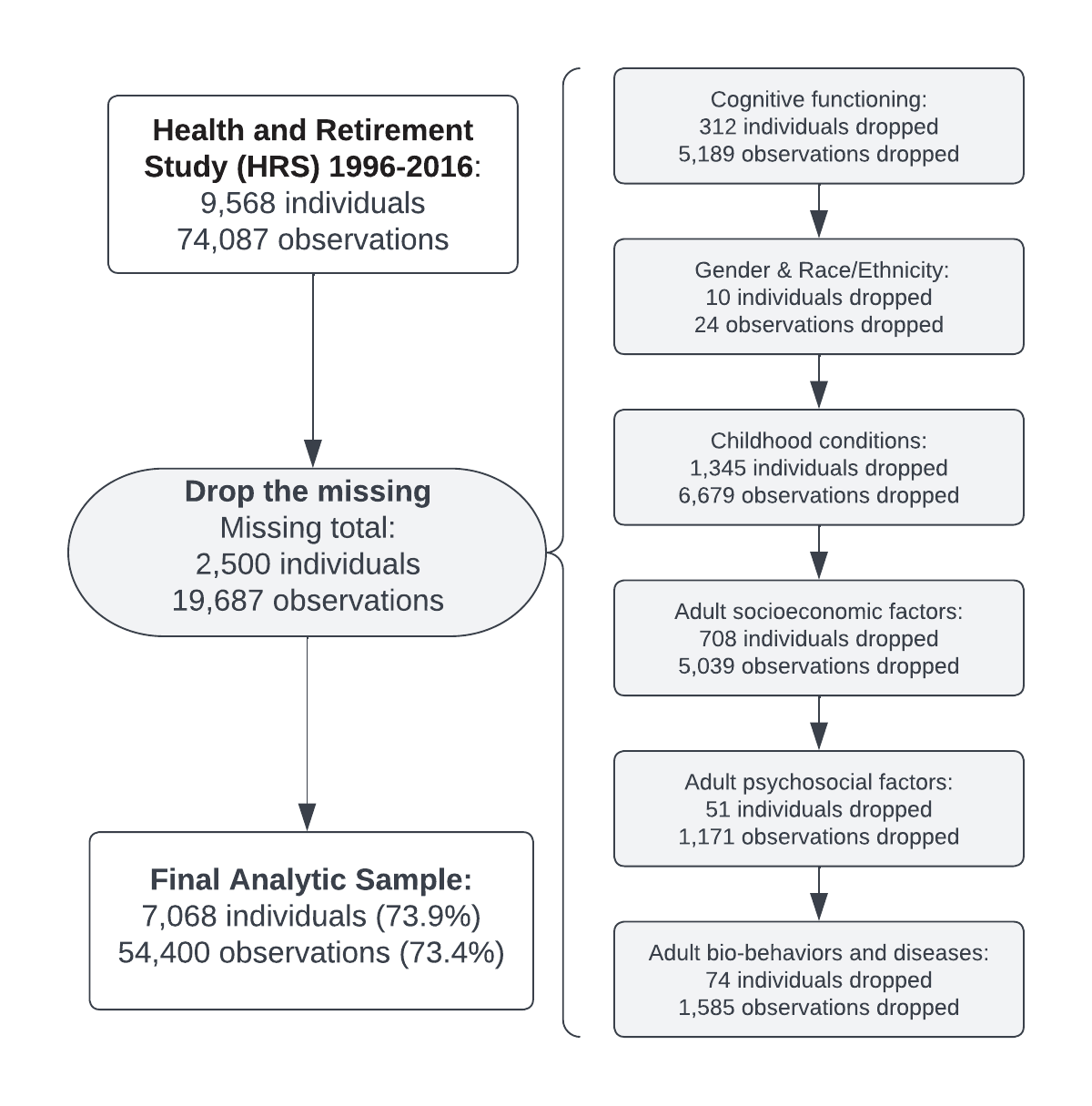
**

**Supporting Information 2. SAS Syntax.**

We used SAS proc mixed package to estimate multilevel growth models of cognitive functioning over ages. Below we provide some sample codes for Tables 2-4.

proc mixed data=temp1 noclprint covtest;

class hhidpn;

model cognitivefunction = /solution ddfm=bw notest;

random intercept/sub=hhidpn;

title "unconditional mean model";

run;

proc mixed data=temp1 noclprint covtest;

class hhidpn;

model cognitivefunction = age_54 age_54s /solution ddfm=bw notest;

random intercept age_54 /sub=hhidpn type=un;

title "unconditional growth model";

run;

***Sample codes for Table 2: what factors explain the variation in intercept and slope**;

proc mixed data=temp1 noclprint covtest;

class hhidpn ragender(ref="0");

model cognitivefunction = age_54 age_54s ragender age_54*ragender /solution ddfm=bw notest;

random intercept age_54 /sub=hhidpn type=un;

title "conditional growth model_gender";

run;

proc mixed data=temp1 noclprint covtest;

class hhidpn rarace(ref="1") rhealthch rafinanch hitotqc hatotaqc rmstatc(ref="1") rmarc(ref="1") rareligc(ref="4")

robesityc(ref="2");

model cognitivefunction = age_54 age_54s ragender rarace rhealthch cheight rafinanch rpeduc raedyrsc roccupc hitotqc hatotaqc

rmstatc rmarc hchildc rareligc rcesdc robesityc rsmokevc rsmokenc rvgxc rcondec radlac riadlzac

age_54*ragender age_54*rarace age_54*cheight age_54*col age_54*roccupc age_54*hitotqc age_54*rmstatc age_54*rareligc

age_54*robesityc age_54*rsmokenc age_54*rcondec age_54*radlac /solution ddfm=bw notest;

random intercept age_54 /sub=hhidpn type=un;

title "conditional growth model_allcontrols";

run;

***Sample codes for Table 4: what factors explain within-individuals non-age-related variation***;

proc mixed data=temp1 noclprint covtest;

class hhidpn hitotq(ref="1");

model cognitivefunction = age_54 age_54s hitotq /solution ddfm=bw notest;

random intercept age_54 /sub=hhidpn type=un;

title "conditional growth model_time-varying household income";

run;

***Table 3: how baseline dementia status and cognitive functioning explain the variation in intercept and slope***;

proc sort;

by dementedc;

run;

data temp2;

set temp1;

where sequence>1;

age_56=ragey_e-56;

age_56s=age_56*age_56;

run;

proc mixed data=temp2 noclprint covtest;

class hhidpn;

model cognitivefunction = age_56 age_56s /solution ddfm=bw notest;

random intercept age_56 /sub=hhidpn type=un;

title "unconditional growth model_1st observation removed";

run;

proc mixed data=temp2 noclprint covtest;

class hhidpn dementedc(ref="0");

model cognitivefunction = age_56 age_56s dementedc age_56*dementedc /solution ddfm=bw notest;

random intercept age_56 /sub=hhidpn type=un;

title "conditional growth model_by demented 1st";

run;

proc mixed data=temp2 noclprint covtest;

class hhidpn;

model cognitivefunction = age_56 age_56s cognitivefunctionc age_56*cognitivefunctionc /solution ddfm=bw notest;

random intercept age_56 /sub=hhidpn type=un;

title "conditional growth model_by cognition 1st";

run;

**Supporting Information 3. Baseline Predictors of Intercepts and Slopes of Change of Cognitive Functioning from Multilevel Linear Growth Models, Health Retirement Study, 1996-2016**

| **Model** | | **Fixed effects** | | **Between-person residual variance in intercept** $\sigma_{0}^{2}$ | **Between-person residual variance in slope** $\sigma_{1}^{2}$ | **Within-person residual variance** $\sigma_{\epsilon}^{2}$ | **BIC** |
| --- | --- | --- | --- | --- | --- | --- | --- |
|  |  | **Intercept** | **Slope**^a^ |  |  |  |  |
| (1) | Unconditional mean model | 15.520 (0.042)*** |  | 10.903 (0.212)*** |  | 8.946 (0.058)*** | 289334 |
| (2) | Unconditional growth model | 17.383 (0.070)*** | -0.068 (0.008)*** | 13.301 (0.346)*** | 0.0179 (0.001)*** | 6.866 (0.048)*** | 280179 |
| **Demographic Characteristics** | | | | | | |  |
| (3) | Men | -1.145 (0.106)*** | 0.024 (0.005)*** | 13.034 (0.340)*** | 0.0178 (0.001)*** | 6.865 (0.048)*** | 280076 |
| (4) | Race (ref=White) |  |  | 11.697 (0.317)*** | 0.0177 (0.001)*** | 6.870 (0.048)*** | 279064 |
|  | Black | -2.932 (0.144)*** | -0.033 (0.008)*** |  |  |  |  |
|  | Hispanic | -3.097 (0.187)*** | 0.011 (0.010) |  |  |  |  |
|  | Others | -1.484 (0.366)*** | 0.001 (0.020) |  |  |  |  |
| **Childhood Conditions** | | | | | | |  |
| (5) | Childhood health (ref=excellent) |  |  | 13.040 (0.342)*** | 0.0179 (0.001)*** | 6.866 (0.048)*** | 280035 |
|  | Very good | -0.468 (0.129)*** | 0.001 (0.001) |  |  |  |  |
|  | Above average | -1.343 (0.146)*** | -0.005 (0.007) |  |  |  |  |
|  | Fair | -0.905 (0.250)*** | -0.026 (0.013) |  |  |  |  |
|  | Poor | -1.770 (0.456)*** | 0.001 (0.023) |  |  |  |  |
| (6) | Adult height | -0.343 (0.530) | 0.070 (0.027)** | 13.309 (0.346)*** | 0.0179 (0.001)*** | 6.866 (0.048)*** | 280176 |
| (7) | Childhood finance (ref= pretty well off) |  |  | 13.121 (0.343)*** | 0.0179 (0.001)*** | 6.867 (0.048)*** | 280093 |
|  | About average | -0.865 (0.230)*** | 0.009 (0.012) |  |  |  |  |
|  | Poor | -1.605 (0.240)*** | 0.005 (0.012) |  |  |  |  |
| (8) | Parental education | 0.321 (0.014)*** | 0.000 (0.001) | 11.895 (0.322)*** | 0.0178 (0.001)*** | 6.872 (0.048)*** | 279377 |
| **Adult Socioeconomic Status** | | | | | | |  |
| (9) | Years of education | 0.601 (0.016)*** | 0.000 (0.001) | 9.944 (0.285)*** | 0.0176 (0.001)*** | 6.876 (0.048)*** | 277955 |
| (10) | College degree | 2.542 (0.128)*** | 0.020 (0.006)** | 12.231 (0.335)*** | 0.0177 (0.001)*** | 6.872 (0.048)*** | 279430 |
| (11) | Occupation (ref=Blue collar) | 2.272 (0.108)*** | 0.018 (0.006)** | 12.116 (0.325)*** | 0.0177 (0.001)*** | 6.869 (0.048)*** | 279347 |
| (12) | Household income (ref=bottom 25%) |  |  | 11.791 (0.318)*** | 0.0176 (0.001)*** | 6.863 (0.048)*** | 279208 |
|  | 25%-75% | 2.269 (0.152)*** | 0.003 (0.008) |  |  |  |  |
|  | 75%+ | 3.623 (0.158)*** | 0.019 (0.009)* |  |  |  |  |
| (13) | Household wealth (ref=bottom 25%) |  |  | 11.818 (0.319)*** | 0.0178 (0.001)*** | 6.863 (0.048)*** | 279227 |
|  | 25%-75% | 2.086 (0.131)*** | 0.006 (0.007) |  |  |  |  |
|  | 75%+ | 3.405 (0.147)*** | 0.019 (0.008)* |  |  |  |  |
| **Adult Psychosocial Factors** | | | | | | |  |
| (14) | Marital status (ref=married) |  |  | 13.223 (0.344)*** | 0.0178 (0.001)*** | 6.866 (0.048)*** | 280131 |
|  | Partnered | -0.678 (0.296)* | -0.008 (0.016) |  |  |  |  |
|  | Separated/divorced | -0.561 (0.156)*** | -0.013 (0.008) |  |  |  |  |
|  | Widowed | -0.623 (0.199)** | -0.022 (0.015)* |  |  |  |  |
|  | Never married | -0.584 (0.299)* | -0.022 (0.015) |  |  |  |  |
| (15) | Number of marriages (ref=1) |  |  | 13.294 (0.346)*** | 0.0179 (0.001)*** | 6.867 (0.048)*** | 280187 |
|  | 0 | -0.569 (0.290)* | -0.021 (0.015) |  |  |  |  |
|  | 2 | -0.115 (0.131) | -0.011 (0.007) |  |  |  |  |
|  | 3 | 0.079 (0.248) | -0.019 (0.013) |  |  |  |  |
|  | 4+ | -0.208 (0.435) | -0.025 (0.023) |  |  |  |  |
| (16) | Number of living children | -0.193 (0.026)*** | 0.000 (0.001) | 13.154 (0.343)*** | 0.0180 (0.001)*** | 6.866 (0.048)*** | 280107 |
| (17) | Religion (ref=none) |  |  | 13.275 (0.346)*** | 0.0178 (0.001)*** | 6.867 (0.048)*** | 280134 |
|  | Protestant | -0.483 (0.263) | -0.031 (0.014)* |  |  |  |  |
|  | Catholic | -0.401 (0.274) | -0.016 (0.014) |  |  |  |  |
|  | Jewish | 0.659 (0.481) | 0.036 (0.025) |  |  |  |  |
|  | Other | -0.297 (0.702) | -0.091 (0.036)* |  |  |  |  |
| (18) | CESD | -0.500 (0.029)*** | -0.004 (0.002)* | 12.416 (0.331)*** | 0.0179 (0.001)*** | 6.867 (0.048)*** | 279635 |
| **Adult Bio-behaviors and Diseases** | | | | | | |  |
| (19) | BMI category (ref=normal weight) |  |  | 13.232 (0.345)*** | 0.0179 (0.001)*** | 6.867 (0.048)*** | 280120 |
|  | Underweight | -1.011 (0.553) | 0.003 (0.031) |  |  |  |  |
|  | Overweight | -0.455 (0.126)*** | -0.002 (0.006) |  |  |  |  |
|  | Class I obese | -0.616 (0.155)*** | -0.016 (0.008) |  |  |  |  |
|  | Class II/III obese | -0.922 (0.218)*** | -0.034 (0.011)** |  |  |  |  |
| (20) | Smoking status (ref=never smoker) |  |  | 13.230 (0.344)*** | 0.0179 (0.001)*** | 6.866 (0.048)*** | 280128 |
|  | Former smoker | -0.256 (0.120)* | 0.004 (0.006) |  |  |  |  |
|  | Current smoker | -0.431 (0.143)** | -0.024 (0.008)** |  |  |  |  |
| (21) | Vigorous activity | 0.420 (0.107)*** | -0.000 (0.005) | 13.264 (0.345)*** | 0.0179 (0.001)*** | 6.866 (0.048)*** | 280166 |
| (22) | Number of chronic diseases | -0.490 (0.045)*** | -0.010 (0.002)*** | 12.936 (0.338)*** | 0.0179 (0.001)*** | 6.863 (0.048)*** | 279883 |
| (23) | High blood pressure | -0.895 (0.110)*** | -0.006 (0.006) | 13.124 (0.342)*** | 0.0179 (0.001)*** | 6.865 (0.048)*** | 280063 |
| (24) | Diabetes | -1.275 (0.174)*** | -0.040 (0.010)*** | 13.101 (0.342)*** | 0.0178 (0.001)*** | 6.864 (0.048)*** | 279998 |
| (25) | Lung disease | -0.744 (0.249)** | 0.006 (0.014) | 13.278 (0.345)*** | 0.0179 (0.001)*** | 6.866 (0.048)*** | 280174 |
| (26) | Heart disease | -0.475 (0.165)** | -0.017 (0.009)* | 13.271 (0.345)*** | 0.0179 (0.001)*** | 6.865 (0.048)*** | 280156 |
| (27) | Stroke | -2.144 (0.321)*** | -0.030 (0.018) | 13.153 (0.343)*** | 0.0179 (0.001)*** | 6.865 (0.048)*** | 280075 |
| (28) | Psychiatric problems | -1.537 (0.202)*** | -0.025 (0.011)* | 13.125 (0.342)*** | 0.0179 (0.001)*** | 6.865 (0.048)*** | 280051 |
| (29) | Cancer | 0.635 (0.221)** | -0.010 (0.011) | 13.284 (0.346)*** | 0.0179 (0.001)*** | 6.866 (0.048)*** | 280178 |
| (30) | Arthritis | -0.584 (0.108)*** | -0.009 (0.005) | 13.218 (0.344)*** | 0.0179 (0.001)*** | 6.867 (0.048)*** | 280121 |
| (31) | ADL | -0.963 (0.086)*** | -0.011 (0.005)* | 12.857 (0.338)*** | 0.0178 (0.001)*** | 6.861 (0.048)*** | 279921 |
| (32) | IADL | -1.574 (0.114)*** | 0.001 (0.007) | 12.665 (0.334)*** | 0.0178 (0.001)*** | 6.861 (0.048)*** | 279874 |
| **All Factors** | | | | | | |  |
| **(33)** | **All factors** |  |  | **8.273 (0.254)***** | **0.0169 (0.001)***** | **6.871 (0.048)***** | **276537** |
| Sample size (number of observations): 7,068 individuals (54,400 observations) | | | | | | |  |

*Note:*

^a^ A quadratic function of age is included in the models. Constrained by the page size, it is omitted from the table.

**p* < .05; ***p* < .01; ****p* < .001
